# Ensilage and Disease Again

**Published:** 1881-10

**Authors:** 


ENSILAGE AND DISEASE AGAIN.—Taking up the suggestion made by us in a previous editorial, that the much-discussed ensilage may cause disease, a writer in the *National Live Stock Journal* makes several points that deserves attention.

In the first place, ensilage is a wet food, containing just about as much water (75 per cent.) as grass. It is a question whether such succulent food is good for animals in the close confinement of winter. Again, there is a danger that the ensilage may preserve anthrax germs.

There may also be various noxious plants packed away with the corn stalks. If so, the conditions of the silo are such as would bring out all their poisonous properties. The dangers from excessive fermentation, and the effect of the new diet upon the fertility of the animal are also to be considered.

Altogether, it is more than ever apparent that the silo is a thing to be watched by the veterinarian.

